# Association of cancer-related mortality, age and gonadectomy in golden retriever dogs at a veterinary academic center (1989-2016)

**DOI:** 10.1371/journal.pone.0192578

**Published:** 2018-02-06

**Authors:** Michael S. Kent, Jenna H. Burton, Gillian Dank, Danika L. Bannasch, Robert B. Rebhun

**Affiliations:** 1 Department of Surgical and Radiological Sciences, University of California Davis School of Veterinary Medicine, Davis, CA, United States of America; 2 Koret School of Veterinary Medicine, The Hebrew University of Jerusalem, Rehovot, Israel; 3 Department of Population Health and Reproduction, University of California Davis School of Veterinary Medicine, Davis, CA, United States of America; Bauer Research Foundation, UNITED STATES

## Abstract

Golden retriever dogs have been reported to have an increased prevalence of cancer compared to other breeds. There is also controversy over the effect spay or neuter status might have on longevity and the risk for developing cancer. The electronic medical records system at an academic center was searched for all dogs who had a necropsy exam from 1989–2016. 9,677 canine necropsy examinations were completed of which 655 were golden retrievers. Age was known for 652 with a median age of death 9.15 years. 424 of the 652 (65.0%) were determined to have died because of cancer. The median age for dying of a cause other than cancer was 6.93 years while those dying of cancer had a median age of 9.83 years (p<0.0001). There was no significant difference in the proportion of intact males and castrated males dying of cancer (p = 0.43) but a greater proportion of spayed females died of cancer compared to intact females (p = 0.001). Intact female dogs had shorter life spans than spayed female dogs (p<0.0001), but there were no differences between intact and castrated males. Intriguingly, being spayed or neutered did not affect the risk of a cancer related death but increasing age did. The most common histologic diagnosis found in golden retrievers dying of cancer was hemangiosarcoma (22.64%) followed by lymphoid neoplasia (18.40%). Overall golden retriever dogs have a substantial risk of cancer related mortality in a referral population and age appears to have a larger effect on cancer related mortality than reproductive status.

## Introduction

Cancer is the leading cause of death in pet dogs, however, both lifespan and the incidence of cancer can vary between breeds[1–8]. Golden Retrievers (GR) have been recognized in several studies to have a higher prevalence of neoplasia than other popular breeds[1, 5, 9–11]. One study using the Veterinary Medical Database (VMDB), determined that neoplasia was the most common cause of death for the majority of purebred dogs with approximately 50% of 4,029 GR reportedly dying of neoplasia which was second only to the Bernese Mountain dog (54.5%)[5]. It is important to note that the cause of death in 25% of GR in that study was unknown or unclassified, therefore, of GR with a reported cause of death nearly 70% were reported to have died from neoplasia. The VMDB is abstracted from medical records at member hospitals at North American veterinary colleges, and therefore the cause of death is not always necropsy confirmed. Craig et al determined that cancer was the cause of death in 56.6% of 297 GR presenting for necropsy between 1985 and 1999; and this was more than German shepherds, Labrador retrievers, or Rottweilers[9]. The most common histologies described in the GR necropsy reports included hemangiosarcoma (31.5%), lymphoma (14.3%), carcinomas (13.1%), other sarcomas (8.9%), tumors of the CNS (7.7%), histiocytic (6.5%), and endocrine or neuroendocrine tumors (3.6%). The average age of death for GR reported in this necropsy study was 8.6 years for neoplasia, 7.3 years for all causes, and 5.7 years for non-neoplastic diseases. This compares similarly with the overall mean age of death for GR in the North American VMDB of 6.6 years, likely reflecting a biased referral population[12].

Interestingly, this data contrasts that of European studies in regard to longevity and incidence of cancer in GR. Of 927 deaths in GR from primary care practices outside of North America, the median age of death was 11.0–12.5 years[1, 8, 13, 14] and only 20–39% of GR deaths were attributed to neoplasia[1, 13, 15]. Based on this data, it appears that GR within the United States (US) may have both a higher incidence of cancer and a shorter lifespan. However, while numerical differences exist between European and US studies, several important and potentially confounding factors have been identified[16]. Differences between such studies include case selection bias between case studies (primary vs. referral hospitals), necropsy confirmed versus client survey studies, and significant geographic differences between spay and neuter practices in the US and Europe[17].

Several studies have now demonstrated an association between spay neuter status or hormonal exposure on the incidence of cancer and longevity in pet dogs[8, 17–20] [21–25]. As per a 2016 survey, 86% of dogs in the United States are spayed or neutered[26]. A recent US study examining over 40,000 dogs found a significant increase in cancer incidence for dogs that were spayed or neutered. This increased incidence still remained when the dogs were broken into age categories [27]. One recent study examined orthopedic disease and cancer specifically in GR and Labrador retrievers, concluding that earlier spay or neuter was associated with increased incidence of orthopedic disease and certain cancers in these breeds. Another recent study reported that gonadectomy was associated with increased risk of cancer, independent of breed[20]. However, neither of these studies reported lifespan. While one prior study reported that necropsy confirmed cancers were overrepresented in US GR prior to 1999, there is some question as to whether the incidence of cancer amongst US GR has continued to increase over the past two decades. Furthermore, this prior report did not identify sex or spay/neuter status amongst GR deaths[9]. We therefore questioned whether the cancer-related mortality in GR at our institution has changed since 1989 and specifically whether overall reproductive status is associated with longevity or cancer in the GR breed.

## Materials and methods

### Case selection

The UC Davis Electronic Medical Record System was searched from 1/1/1989 through 12/31/2016 for all GR dogs evaluated and for all dogs undergoing necropsy examination at the UC Davis Veterinary Medical Teaching Hospital (VMTH). 1989 was chosen as the start date of this study as this is when the electronic medical record system began incorporating clinical data and necropsy results. For dogs undergoing necropsy exam the age, birth date, sex, breed, date of necropsy examination and pathological diagnoses were recorded. If age was not known then the cases were excluded. Age was calculated from date of birth to date of necropsy. If only the birth month and year were known, the date of birth was assigned as the 15^th^ day of that month; if only the birth year was known then the birth month and day was assigned as June 30^th^. Each pathologic diagnosis found on necropsy for each patient was reviewed and coded to indicate if the dog was diagnosed with cancer but died of an unrelated cause or if the dog’s cause of death was attributable to cancer. For those dogs whose death was attributed to cancer the type of cancer was also categorized as lymphoid, hemangiosarcoma, osteosarcoma, other sarcoma, carcinoma, meningioma, histiocytic, pituitary origin, melanoma or other. Tumor histologies included in the other sarcoma, carcinoma and other categories are presented in S1 Table.

### Statistical analysis

Descriptive statistics were done. Age was evaluated for normality using the Shapiro-Wilk method and Mann-Whitney tests were done to explore association between age and reproductive status. To look for differences in proportions between categorical variables including sex, reproductive status, tumor histology, and death attributable to cancer Chi-squared tests were used unless there were less than 6 individuals in a group in which case a fisher’s exact test was used. To test whether the there was a change in time over the proportion of GR undergoing a necropsy exam versus the total number of GR seen during the same time period linear regression was done. Logistic regression was done and odds ratio were calculated to look for the effect of age, sex and reproductive status on cancer related deaths. Statistics were done using a commercially available software program (Stata 14). A p value < 0.05 was considered statistically significant.

## Results

**Fig 1:**
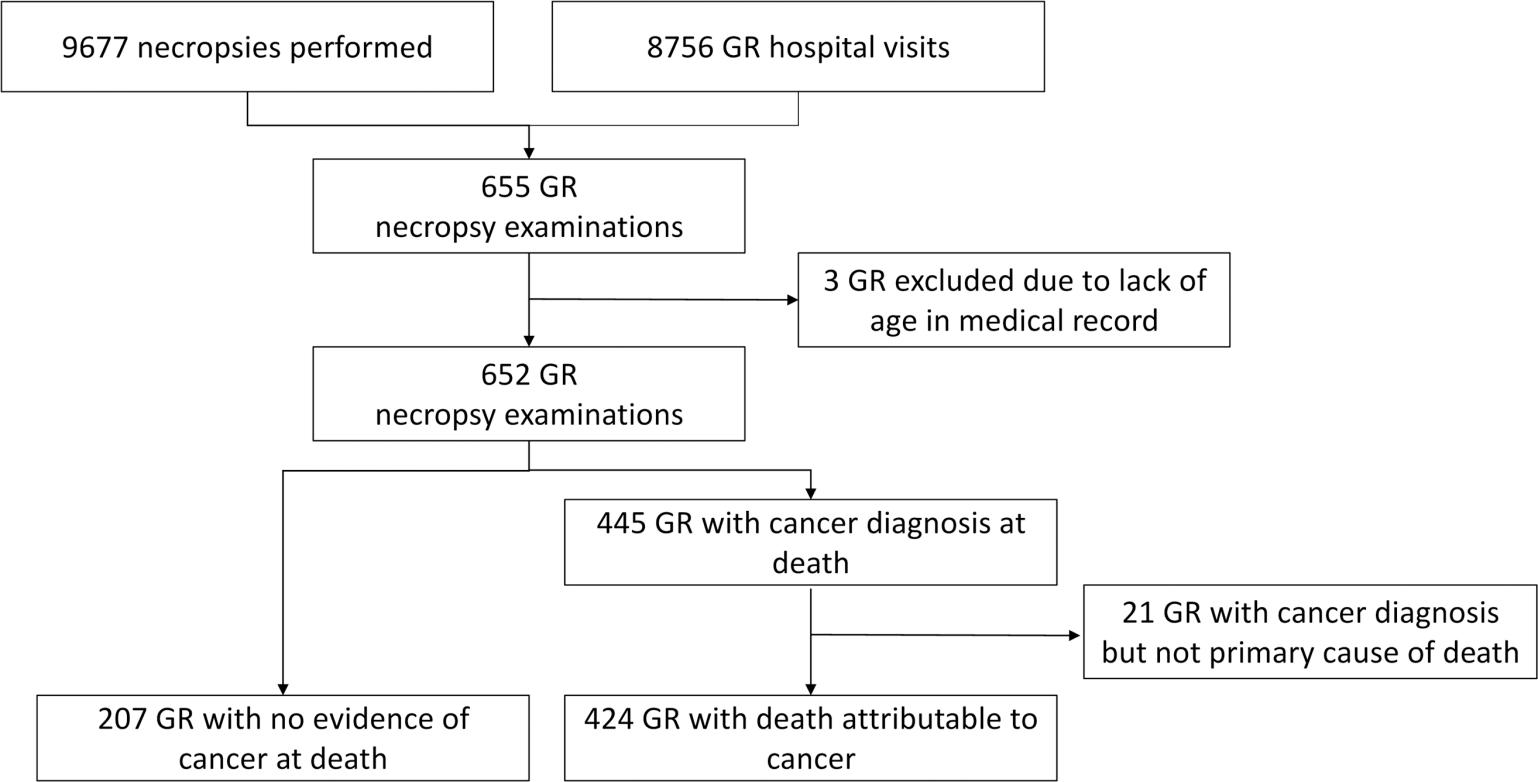
Study schema. Study schema showing cases included from 1989–2016 from an academic teaching hospital. GR = golden retriever dog.

**Fig 2:**
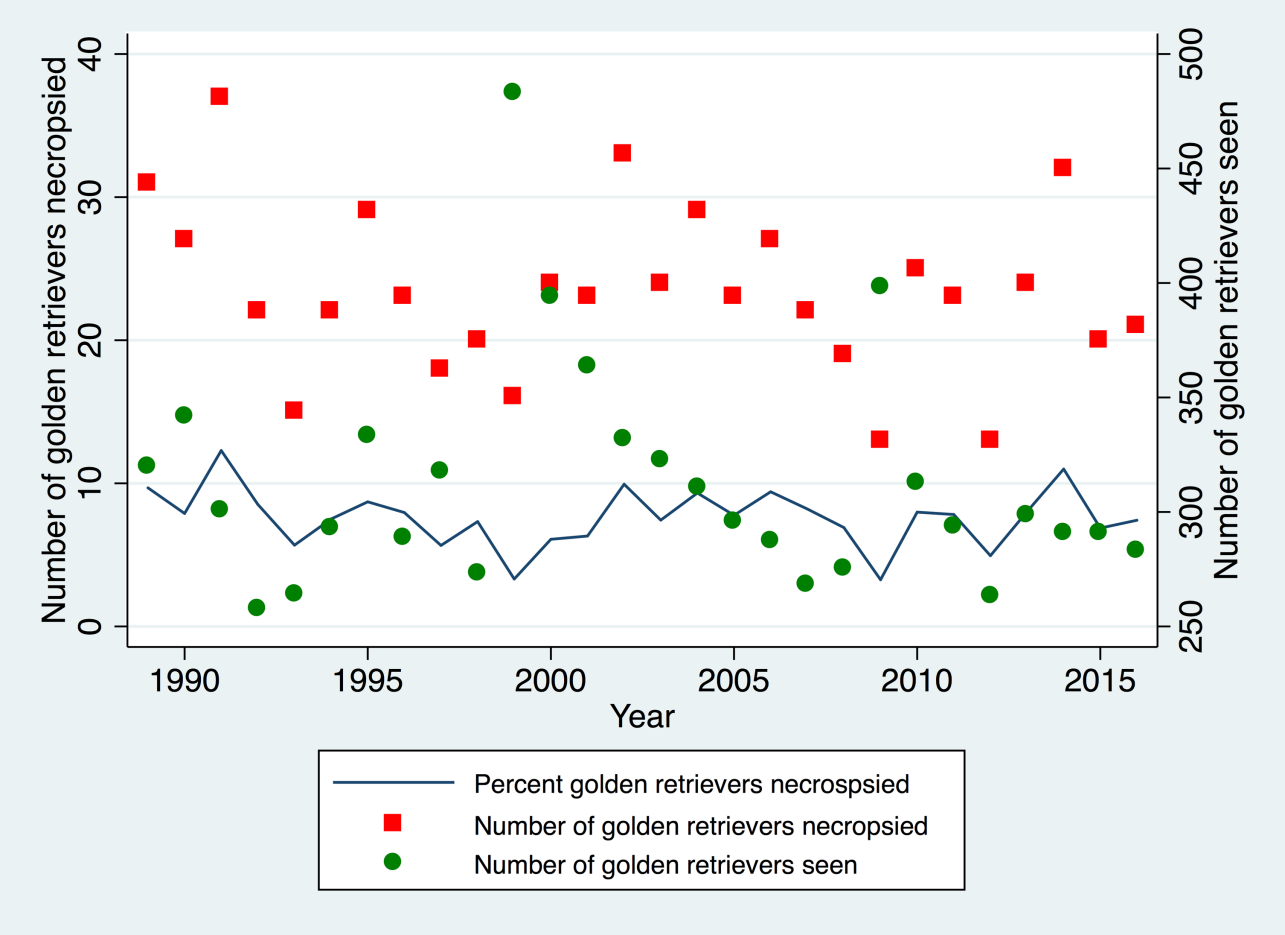
Golden retriever dogs seen and undergoing necropsy. Scatter plot showing in green circles the total number of golden retrievers per year undergoing necropsy exam and in red squares the total number of golden retrievers seen in the hospital per year. The solid line represents the percentage of golden retrievers undergoing necropsy divided by the total golden retriever animals seen that year.

A total of 8,756 individual GR were evaluated at the VMTH during the study period. During this same time period, 9,677 canine necropsy examinations were completed of which 655 were GR. Age was known for 652 GR. The schema for the study is presented in Fig 1 and the data table is presented in S1 Dataset. The yearly totals for necropsy examinations on GR are shown in Fig 2. There was no change in the proportion of GR dogs having a necropsy exam to the number of GR dogs seen in the hospital over time (p = 0.47).

**Fig 3:**
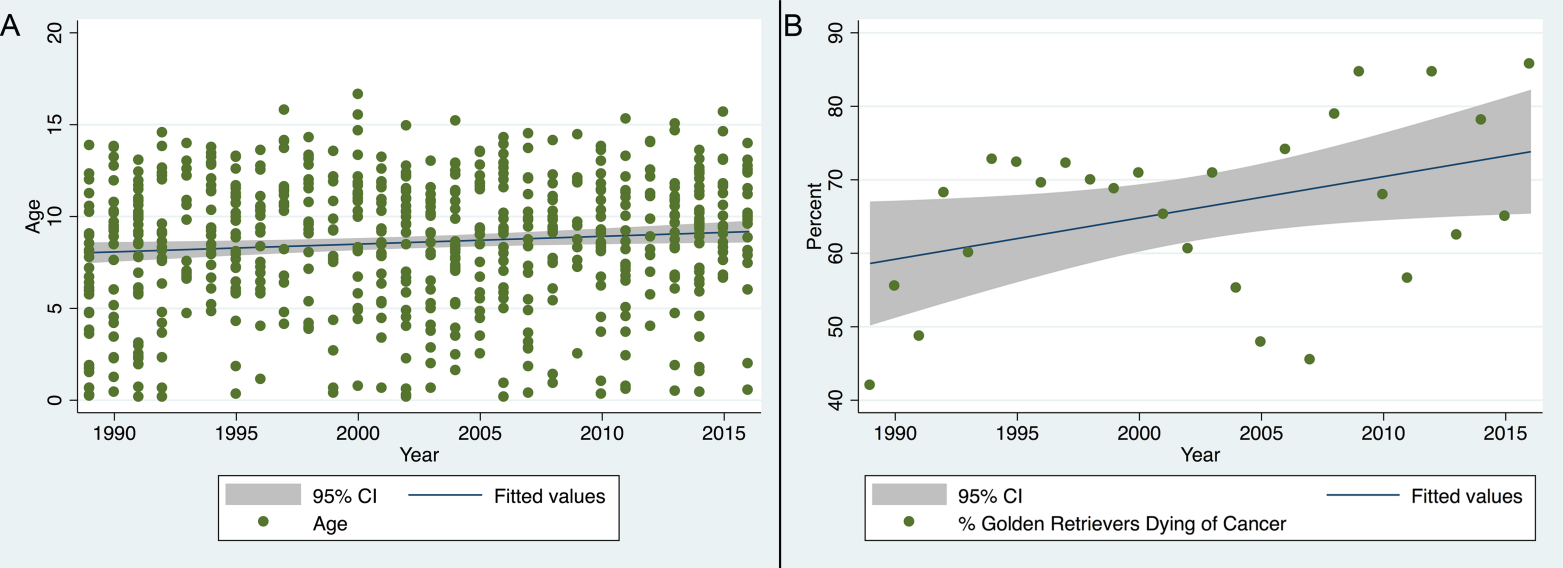
Percent of golden retriever dogs dying of cancer and age of death over time. (A) Scatter plot with fitted regression line and 95% CI showing percent of golden retriever dogs undergoing necropsy exam that died from cancer by year. The results show a significant (P = 0.38, R^2^ = 0.16) but weak increase in the proportion of dogs with a cancer diagnosis over time. (B) Scatter plot with fitted regression line and 95% CI showing age of golden retriever dogs presenting for necropsy over time. The results show a significant but week increase in age over time (P = 0.01, R2 = 0.009).

Based on necropsy findings, 445 of the 652 (68.3%) GR undergoing necropsy examination were diagnosed with some form of cancer and 424 of the 652 (65.0%) GR were determined to have died or have been euthanized because of cancer. We then evaluated if the proportion of GR dying of cancer changed over time. There was a statistically significant but weak increase in the proportion of dogs dying from cancer over time (P = 0.038, R2 = 0.16; Fig 3A). Similarly, we found a statistically significant but weak association between the age of GR necropsied increasing over time (P = 0.01, R2-0.009; Fig 3B).

**Fig 4:**
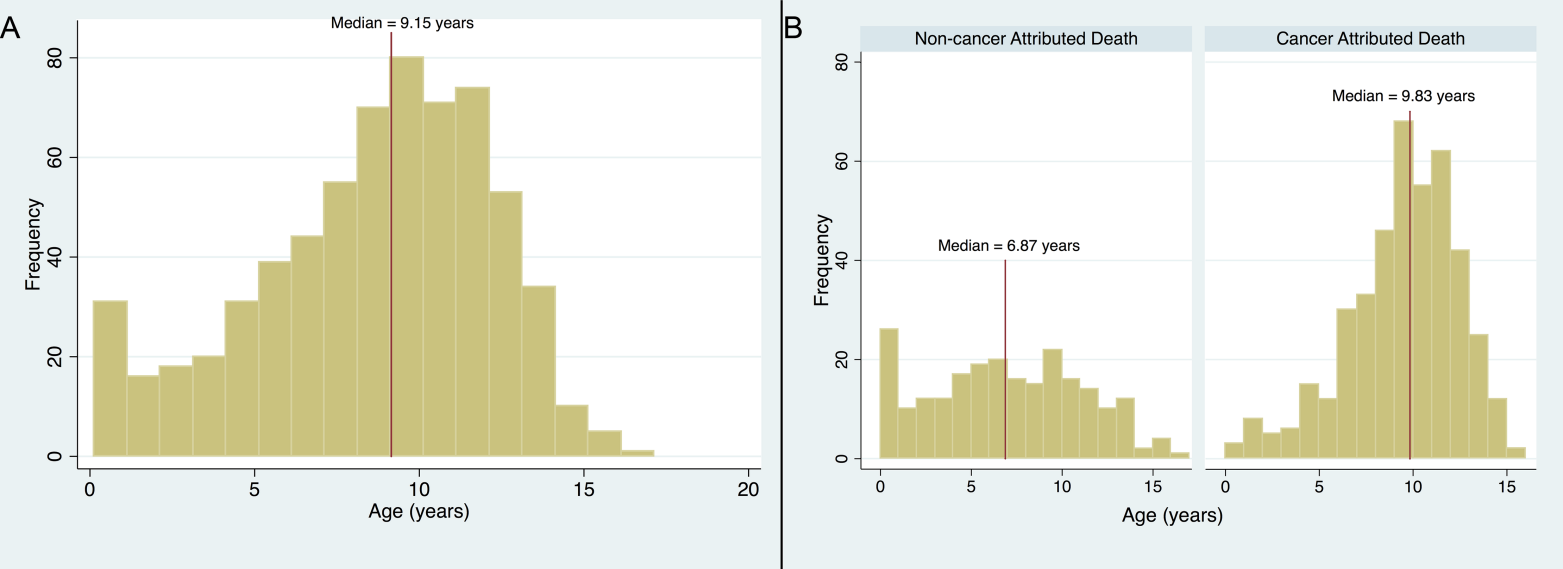
Age distribution of golden retriever dogs. (A) Histogram of age for all golden retriever dogs undergoing a necropsy exam with a median age of 9.15 years. (B) Histogram of ages of all golden retriever dogs undergoing a necropsy exam categorized by having a death attributable to cancer or not with a median age of death for those dogs dying of non-cancerous cause of 6.87 years and a median age of death of 9.83 years for those dogs with a cancer attributable death.

The median age of death for all GR having had a necropsy exam during the study time period was 9.15 years (Range 0.13–16.62). The age distribution for all GR is shown in Fig 4A. Age was not found to be normally distributed. The median age for GR dying of a cause other than cancer was 6.93 years (range 0.1–16.6 years) while those dying of cancer had a median age of 9.83 years (range 0.4–15.5 years) (Fig 4B). This difference was statistically significant (p<0.0001). The overall odds ratio of dying of cancer increased with age and was 1.23 (95% CI 1.17–1.29; P < 0.0001). When categorizing the cases by the interquartile ranges, there was an increase in the odds ratio dying of a cancer related cause through the first quartile of age (Age < 7.86 years, OR 1.33 95% CI 1.18–1.51, p<0.0001). This risk remained constant through the next two quartiles (ages 7.86–9.83: OR 0.92 95% CI 0.47 and 1.79, p = 0.80 and 9.83–11.58: OR 1.01 95% CI 0.45–2.27 p = 0.98) and then decreased again with after age 11.58 (OR 0.63 95% CI 0.44–0.91 p = 0.01).

There were a total of 118 intact males (18.1%), 228 castrated males (35.0%), 58 intact females (8.9%) and 248 spayed females (38.0%) GR in the study. In 77 (65.3%) of intact males, cancer was attributed as the cause of death on necropsy examination. One hundred fifty-eight (69.3%) neutered males had had deaths attributable cancer. Twenty-four (41.4%) intact females died of cancer and 165 (66.5%) spayed female had a death attributed to cancer. There was no significant difference in the proportion of intact males and castrated male GR dying of cancer (p = 0.43) but there was a significant difference in the proportion of intact to spayed female GR dying of cancer (p = 0.001), with a greater proportion of spayed females dying of cancer.

**Fig 5:**
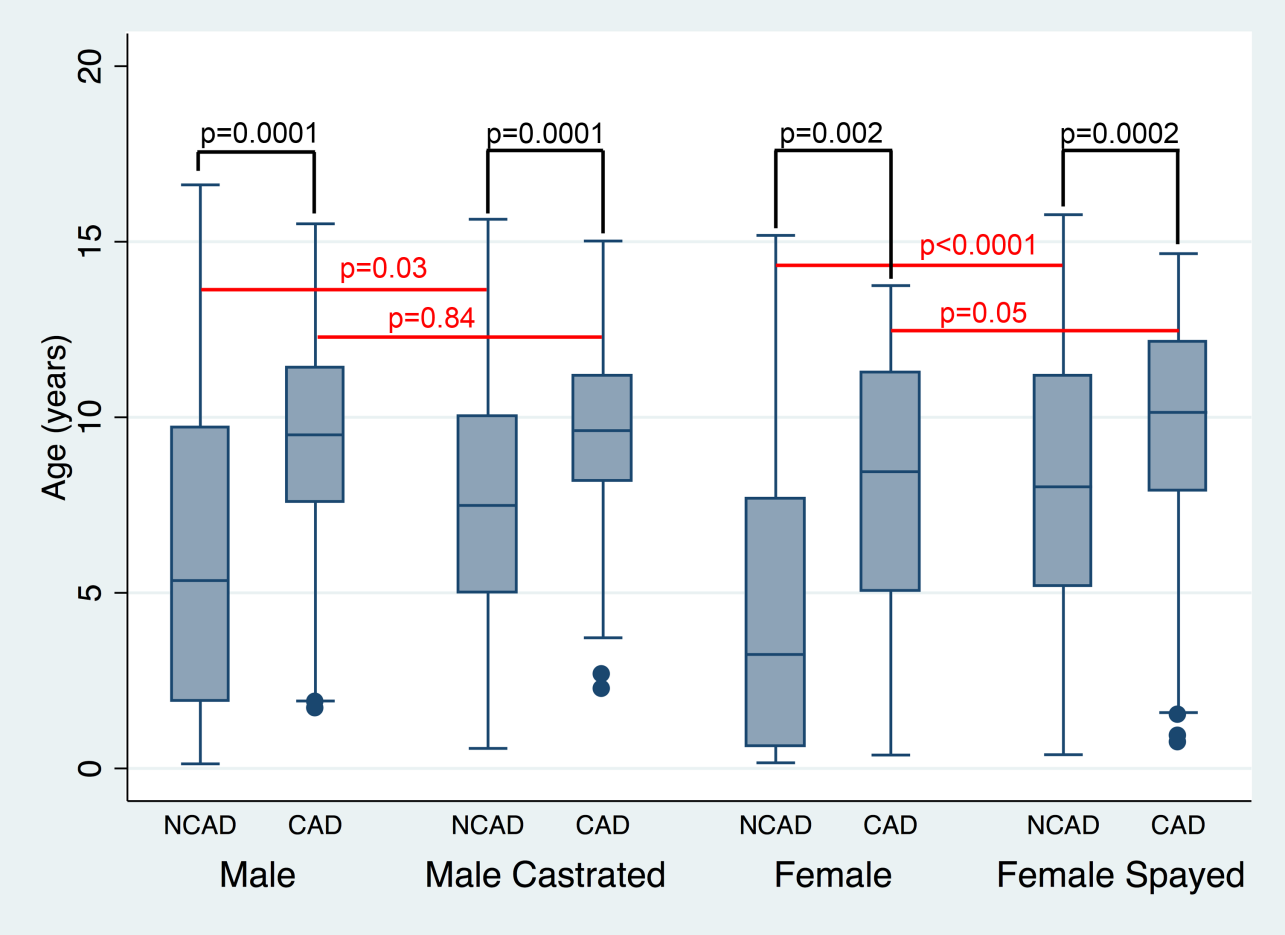
Age of golden retrievers broken out by sex and cause of death. Box and whisker plot showing median age, interquartile range, adjacent values and outliers of golden retriever dogs who underwent a necropsy exam and an attributable or non-attributable death from cancer categorized by sex and spay or neuter status. P values are shown for differences between cancer and non-cancer attributed deaths within each sex category and for differences between sex category within the cancer and non-cancer attributed death groups. NCAD = non-cancer attributable death, CAD = Cancer attributable death.

The median age for GR presenting for necropsy examination was 8.68 years (range 0.13–16.62 years) for intact males and 9.35 years (range 0.57–15.64 years) for male castrated GR. There was no significant difference between these ages (p = 0.15). The median age for intact female GR presenting for necropsy was 5.89 years (range 0.16–15.18 years) and for female spayed GR was 9.51 years (range 0.39–15.77 years; Fig 5). This difference was significantly different (p<0.0001).

For those GR not dying of cancer, the median age of death was 5.35 years (range 0.13–16.62 years) for intact male GR, 7.49 years (range 0.57–15.64 years) for male castrated GR, 3.25 years (range 0.16–15.18 years) for intact females and 8.0 years (range 0.39–15.77 years) for female spayed GR. For those GR dying of cancer, the median age of death for intact male dogs was 9.5 years (range 1.71–15.51 years), for male castrated dogs was 9.62 years (range 2.25–15.02 years), for intact female dogs was 8.45 years (range 0.38–13.75 years) and for female spayed dogs was 10.14 years (range 0.73–14.66 years) (Fig 5). The difference in age between those with a cancer-related mortality and non-cancerous causes of mortality remained significant when looking within each sex category (intact male p = 0.0001, male castrated p = 0.0001, intact female p = 0.002 and female spayed p = 0.0002) indicating that those dogs dying of cancer lived longer than those dying of non-cancerous causes.

To further characterize the effect of age and sex on death related to cancer, we explored whether there was a difference in age between intact males and castrated males and intact females and spayed females within the attributed cause of death groups. There was a significant difference in age between intact and castrated male GR that did not die of cancer (P = 0.03) but no difference in ages between intact and castrated male GR that died of a cancer related cause (p = 0.84). In the non-cancer related mortality group there was a significant difference in age between intact and spayed female GR (p<0.0001) but there was no significant difference between the ages of intact and spayed female GR who died of cancer (p = 0.05). Increasing age was found to be significant within each reproductive status group for increasing the odds of being in the cancer related mortality group (Intact male: OR 1.26, 95% CI 1.12–1.41, P<0.0001; Neutered male OR 1.24 95% CI 1.12–1.38, p <0.0001; Intact female OR 1.23 95% CI 1.07–1.41, P = 0.003; Spayed female OR 1.17 95% CI 1.08–1.27, p<0.0001). We also looked at the effect that age and sex and reproductive status had on the odds of death due to a cancer related cause. Logistic regression was done looking at both reproductive status and age as independent variables. In male GR, reproductive status was not found to be significant (OR 0.99 95% CI 0.59–1.64, p = 0.96) while increasing age was (OR 1.25 95% CI 1.16–1.35, P<0.0001). Similarly, in female GR reproductive status was not significant (OR 1.73 95% CI 0.91–3.31, p = 0.09) while increasing age remained a significant factor in the likelihood of being in the cancer related mortality group (OR 1.19 95% CI 1.11–1.27, P<0.0001).

The analysis was repeated for dogs 1 year of age or older to ensure the results were not biased by dogs dying of disease before in theory they could be spayed or neutered and to account for any congential diseases that would potentially increase early mortality and owner reluctance to spay or neuter. There were a total of 623 GR one year of age or older in this truncated study group. 421 GR had a death attributable to cancer. There were 109 intact males, 223 catrated males, 47 intact females and 244 spayed females in the study that were one year of age or older. Seventy-seven (70.6%) intact males, 158 (71.0%) castrated males, 23 (48.9%) intact females and 163 (66.3%) female spayed GR died of cancer. There were no differences between intact and castrated male GR as to the proportion dying of cancer (p = 0.95). There were however differences between the proportion of intact and spayed female GRs dying of cancer (p = 0.024). The median age of this group of GR undergoing necropsy was 9.32 years (range 1.02–16.62). The median age for intact males was 9.04 years (range 1.71–16.62). The median age for castrated males was 9.39 (range 1.79–15.64). The age of intact and castrated males presenting for necropsy was not different (p = 0.53). The median age for intact females was 7.68 years (range 1.02–15.18) and 9.66 years (range 1.25–15.77). These ages were significantly different with intact females dying younger than spayed females (p = 0.0004).

The most common histologic diagnosis found in GR dying of cancer was hemangiosarcoma (n = 96, 22.64% of cases) followed by lymphoid neoplasias (n = 78, 18.40%), carcinomas (n = 55, 12.97%), other sarcomas (n = 42, 9.91%), other cancers (n = 38, 8.96%), meningioma (n = 37, 8.73%), histiocytic (n = 33, 7.78%), osteosarcoma (n = 29, 6.84%), melanoma (n = 9, 2.12%) and pituitary tumor (n = 7, 1.65%). Details of histologic type and frequencies for the carcinomas, other sarcomas and other cancer groups are presented in S1 Table.

**Fig 6:**
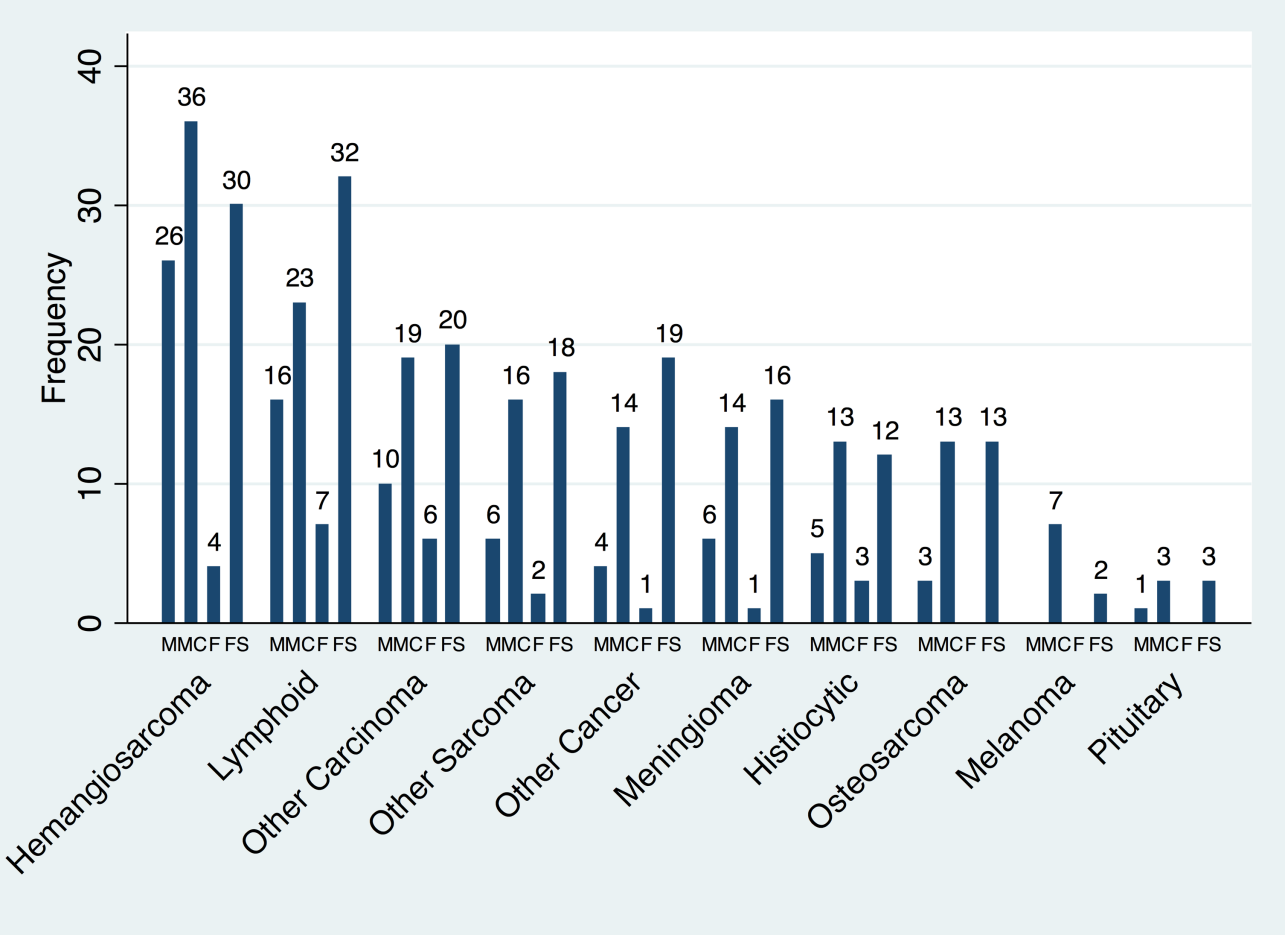
Histologic types of cancer diagnosed by sex. Bar graph showing frequency of tumor type leading to death in golden retriever dogs categorized by sex and spay or neuter status. M = intact male, MC = castrated male, F = Intact female, FS = spayed female.

The frequency for sex distribution for each tumor type is presented in Fig 6. We further examined if the proportion of dogs dying of a particular tumor type was different between intact and castrated male GR or between intact and spayed female GR with the results presented in Table 1.

**Table 1:** Age by tumor histology and comparison of sex groupings.

Tumor histology	Median age in years (range)	P value for comparing proportion of male versus male castrated by histology	P value for comparing proportion of female vs female spayed by histology
Hemangiosarcoma	10.02 (2.65–14.66)	0.18	0.35
Lymphoid neoplasia	8.39 (1.59–14.28)	0.33	0.88
Carcinomas	10.28 (4.27–14.91)	0.96	0.56
Other sarcomas	9.09 (0.38–13.85)	0.49	0.39
Other cancers	10.20 (0.73–15.51)	0.32	0.14
Meningioma	10.78 (4.97–14.61)	0.70	0.21
Histiocytic	9.01 (2.34–13.05)	0.80	1.00
Osteosarcoma	9.26 (1.52–14.5)	0.28	0.14
Melanoma	11.64 (9.65–14.46)	1.00	1.00
Pituitary tumor	9.39 (7.11–12.52)	1.00	1.00

## Discussion

Overall we found that 68.3% GR undergoing necropsy examination were diagnosed with some form of cancer and 65.0% of GR had a death attributable to cancer. This finding is similar or slightly higher than other reported academic studies from the United States[5, 9] and higher than the rates reported from European studies, whose cases were largely from a more general population[1, 3]. One strength of this study is that by virtue of only including cases that had a necropsy exam performed, the pathologic conditions at the time of death are confirmed. We also found a weak but increasing proportion of GR over time in the number of cases of cancer, which could account for some of the difference in the increased percentage of GR dying of cancer compared to older studies.

The median age of death for all GR having had a necropsy exam was 9.15 years. This is less than three European studies that reported a median survival for GR of 12.5, 12.25 & 12.25 years[1, 3, 14]. These studies were done from a general population of GR and the lower median age of death found in our study may be due to the referral nature of our practice[16]. A necropsy study from another academic institution did not report a median age of survival but reported an average age of death for GR of 7.3 years[9]. Another referral practice necropsy study covering the years 1962–1976 reported a mean age of death for GR of 6.7 years[2]. Similarly, the median age at death for GR from the VMDB, in a study covering 1980–1990 reported a median age of death for GR of 6.6 years[12]. This latter study removed from age analysis all dogs dying of trauma or toxic causes if they died at less than one year of age[12]. When analyzing the current data set by removing all dogs that died at one year of age or less the median survival in the current study was 9.32 years of age. While these measures of age of death are not directly comparable to the current study, the cause for the apparent longer survival in our study is unknown, although it is possible that this represents a change in breed longevity or more effective treatment options over time.

The median age for GR dying of a cause other than cancer was 6.93 year (range 0.1–16.6 years) while those dying of cancer had a median age of 9.83 years. The median ages of GR that had a death attributable to cancer were significantly older than those that did not die of cancer. We also found an increase in the odds of dying of cancer with increasing age. In humans the age distribution of death due to cancer increases with age peaking between the ages of 75–84 and then decreases after that[28]. We observed a similar pattern with a peak in the age distribution at around 10 years of age and decreases after this. Overall, dogs dying of disease other than cancer die at a younger age in this population.

Multiple studies have studied the association of neutering and spaying on the development of cancer and survival[17, 25, 29], however, this is a complex issue and determining causation and elucidation of the possible protective mechanism of sex hormonal exposure is challenging in the retrospective studies performed to date. Age is also likely to play an important factor; since cancer deaths peak at a relatively late age in GR, if an individual does not live to an older age cancer is less likely to be the cause of death. This should be viewed in light of our findings that while there were no differences in the age of death between intact and neutered male GR, intact female dogs (5.89 years) lived substantially shorter than spayed female dogs (9.51 years). We should also note that the sample size of intact females was limited which could have affected our outcomes.

There was a significant difference in age between intact and castrated male GR that did not die of cancer with neutered GR dying at an older age, but no difference in age was observed between intact and neutered male GR dogs that died of cancer. For female dogs not dying of cancer, intact female dogs died younger than female spayed dogs, while there was no significant difference in age between intact and spayed females that died of cancer.

These results indicate that dogs who died of cancer realted casues were older than those not dying of non-cancerous causes regardless if they were spayed or neutered. This is supported by our findings that increasing age increased the odds of cancer related mortality when looking within each reproductive status group and when modeling age and reproductive status within female and male groupings.

Our results differ from those found by others using patient records from the same institution[29, 30], however, this is no doubt reflective of differences in study design. Dogs in this study all had histopathologically confirmed diagnoses. Furthermore, the current study included dogs of all ages, while the previous studies truncated the population to only dogs>1 and <9 years of age. If we had only included dogs less than 9 years in the current study we would have excluded 350 of the 655 (53.4%) cases, including 56 (47%) intact males, 131 (57.2%) neutered males, 17 (41%) intact females and 146 (58.4%) of spayed female GR.

Similarly to our study the work of Hoffman et al found that gonadectomy was associated with increased longevity (27). A potential weakness in both of these studies is that these studies did not examine lifetime exposure to hormones as a factor as the work that Waters et al has done (25). The Waters paper found a protecitve effect of gonadal exposure for longevity in long lived female Rottweilers. While the Waters study examined a different dog breed, which could explain some of the differences between their findings and the current study, they limited the population to those dogs living >30% above the average age of the population. In another study this same group discounted the effect of pyometra and mammary cancer on logevity in this breed (24) although, other disease such as infectious disease could account for differences in longevity in the overall population as was found in the Hoffman study (27). This again highlights the complexity of determining the underlying causes of longevity within a single breed of dog, much less all dogs in general.

One weakness of this study is that the timing of spay or neuter was not recorded for most cases in the medical record so we were unable to evaluate the association that hormonal exposure might have on the risk of longevity or dying of cancer. Furthermore, as we are only assessing cancer as a cause of death, we did not evaluate spay or neuter status with the risk of cancer development. Since we did not have access to complete veterinary medical records from referring veterinary practices, it is certainly feasible that some GR in the non-cancer related mortality group had a previous cancer diagnosis that was appropriately treated, never recurred and was not associated with a life-limiting event. One reason for repeating our analysis including only dogs that died at > 1 year of age was to help account for cases that might have been neutered or spayed had they lived to greater than one year of age.

Similarly to a previously published academic study we found that GR dying of cancer most frequently were diagnosed with hemangiosarcomas, lymphoma and carcinomas[9]. We also found GR dying of other sarcomas, meningiomas, histiocytic tumors, osteosarcomas, melanomas and pituitary tumors. While it is not possible to evaluate if GR are at an increased risk for developing these tumor types compared to other breeds from this dataset, several other studies have evaluated this. [3, 31, 32].

As the current study only included necropsy cases, tumor histologies that may occur in GR with an increased risk but that do not lead to death may not be captured. For example, Dobson et al reported the GR dog to be at increased risk of developing a mast cell tumor[3]. This type of tumor was included in the other tumor type category in this study as only 6 dogs had a mast cell tumor at the time of necropsy examination.

Interestingly we found no differences in the proportion of dogs developing any single histology of tumor studied between intact male and neutered male dogs or intact female and female spayed dogs. Again, these findings differ from previous studies.

## Conclusions

Our study shows that GR have a substantial risk of cancer related mortality in a referral population. We found significant differences in lifespan between spayed and intact female dogs, with intact dogs having shorter overall lifespans. We also found that being spayed or neutered did not negatively affect the risk of having a cancer related death. This study highlights the complexity in determining the effect spay or neuter has on the risk of cancer death. As there remain conflicting results between studies as to factors that affect both survival and the risk of developing cancer in dogs, prospective cohort studies are needed to answer these questions, such as the ongoing golden retriever life time study currently being carried out[33].

## Supporting information

S1 Table Tumor histologies and frequencies included in grouped categories. (DOCX)

S1 Dataset Dataset of golden retrievers undergoing necropsy exam. (XLSX)
