# Supplementary material for: Association of cancer-related mortality, age and gonadectomy in golden retriever dogs at a veterinary academic center (1989-2016)
Source: PLoS One. 2018 Feb 6;13(2):e0192578. doi: 10.1371/journal.pone.0192578 (PMC5800597; doi:10.1371/journal.pone.0192578)
Supplement: S1 Table — (DOCX) [file pone.0192578.s001.docx]

| **Diagnosis** | **Number** |
| --- | --- |
| **Carcinomas** | |
| Adenocarcinoma unknown origin | 2 |
| Adrenal adenocarcinoma | 1 |
| Adrenocortical carcinoma | 1 |
| Bronchiolar carcinoma, lung | 4 |
| Carcinoma undifferentiated primary unknown | 3 |
| Cholangiocarcinoma | 2 |
| Choroid plexus carcinoma | 6 |
| Circum-anal gland adenocarcinoma | 1 |
| Gastric adenocarcinoma | 1 |
| Hepatocellular carcinoma | 2 |
| Mammary carcinoma | 2 |
| Mammary gland tumor comedocarcinoma | 1 |
| Nasal adenocarcinoma | 8 |
| Neuroendocrine carcinoma | 3 |
| Ovarian papillary cyst adenocarcinoma, metastatic | 1 |
| Pancreatic adenocarcinoma | 2 |
| pancreatic islet cell tumor | 2 |
| Papillary carcinoma, lung | 2 |
| Renal Carcinoma | 1 |
| Squamous cell carcinoma, nasal planum | 1 |
| Thyroid follicular carcinoma | 1 |
| Thyroid carcinoma c-cell | 1 |
| Transitional cell carcinoma, Bladder | 1 |
| Transitional cell carcinoma, prostate | 4 |
| **Other Sarcomas** | |
| Chondrosarcoma | 3 |
| Fibrosarcoma | 4 |
| Hemangiopericytoma | 1 |
| Leiomyosarcoma | 2 |
| Liposarcoma | 1 |
| Multilobular tumor of bone | 3 |
| Myxosarcoma | 1 |
| Nasal fibrosarcoma | 1 |
| Nerve sheath tumor, brachial plexus | 1 |
| Peripheral nerve sheath tumor, brain | 1 |
| Rhabdomyosarcoma | 2 |
| Sarcoma | 4 |
| Sarcoma, anaplastic | 3 |
| Sarcoma, undifferentiated | 7 |
| Schwannoma, brachial plexus | 1 |
| Soft tissue sarcoma grade 2 | 1 |
| Spindle cell sarcoma | 5 |
| Synovial cell sarcoma | 1 |
| **Other Cancers** | |
| Astrocytoma | 3 |
| Brunner's gland tumor | 1 |
| Carcinoid, metastatic | 1 |
| Carotid Body Tumor | 1 |
| Chemodectoma | 3 |
| Choroid plexus papilloma | 3 |
| Ependymoma | 2 |
| Gastrointestinal stromal tumor | 2 |
| Granular cell tumor, brain | 1 |
| Hamartoma, brain | 1 |
| Hemangioma, brain | 1 |
| Mast cell tumor | 6 |
| Medulloblastoma | 1 |
| Mesothelioma | 4 |
| Nephroblastoma | 1 |
| Oligodendroglioma | 2 |
| Pheochromocytoma | 3 |
| Synovial myxoma | 1 |
| Thymoma | 2 |
